# Assessing behavioral economic biases among young adults who have increased likelihood of acquiring HIV: a mixed methods study in Baltimore, Maryland

**DOI:** 10.1186/s12981-023-00521-3

**Published:** 2023-05-07

**Authors:** Larissa Jennings Mayo-Wilson, Jessica Coleman Lewis, Sarah MacCarthy, Sebastian Linnemayr

**Affiliations:** 1 Department of Health Behavior, Department of Maternal and Child Health, University of North Carolina at Chapel Hill Gillings School of Global Public Health, 310 Rosenau Hall CB #7400, 135 Dauer Drive, Chapel Hill, NC 27599 USA; 2 Department of International Health, Social and Behavioral Interventions Program, Johns Hopkins Bloomberg School of Public Health, 615 N. Wolfe Street, Baltimore, MD 21205 USA; 3 Department of Health Behavior, University of Alabama at Birmingham School of Public Health, 1665 University Boulevard, Birmingham, AL USA; 4 RAND Corporation, 1776 Main Street, Santa Monica, CA 90401 USA

**Keywords:** Behavioral economics, Young adults, Baltimore, African-American, HIV, Prevention, Qualitative, Homelessness, Mixed methods

## Abstract

**Background:**

Behavioral economic (BE) biases have been studied in the context of numerous health conditions, yet are understudied in the field of HIV prevention. This aim of this study was to quantify the prevalence of four common BE biases—present bias, information salience, overoptimism, and loss aversion—relating to condom use and HIV testing in economically-vulnerable young adults who had increased likelihood of acquiring HIV. We also qualitatively examined participants’ perceptions of these biases.

**Methods:**

43 participants were enrolled in the study. Data were collected via interviews using a quantitative survey instrument embedded with qualitative questions to characterize responses. Interviews were transcribed and analyzed using descriptive statistics and deductive-inductive content analyses.

**Results:**

56% of participants were present-biased, disproportionately discounting future rewards for smaller immediate rewards. 51% stated they were more likely to spend than save given financial need. Present-bias relating to condom use was lower with 28% reporting they would engage in condomless sex rather than wait one day to access condoms. Most participants (72%) were willing to wait for condom-supported sex given the risk. Only 35% knew someone living with HIV, but 67% knew someone who had taken an HIV test, and 74% said they often think about preventing HIV (e.g., high salience). Yet, 47% reported optimistically planning for condom use, HIV discussions with partners, or testing but failing to stick to their decision. Most (98%) were also averse (*b* = 9.4, SD  ±.9) to losing their HIV-negative status. Qualitative reasons for sub-optimal condom or testing choices were having already waited to find a sex partner, feeling awkward, having fear, or not remembering one’s plan in the moment. Optimal decisions were attributed qualitatively to self-protective thoughts, establishing routine care, standing on one’s own, and thinking of someone adversely impacted by HIV. 44% of participants preferred delayed monetary awards (e.g., future-biased), attributed qualitatively to fears of spending immediate money unwisely or needing time to plan.

**Conclusion:**

Mixed methods BE assessments may be a valuable tool in understanding factors contributing to optimal and sub-optimal HIV prevention decisions. Future HIV prevention interventions may benefit from integrating savings products, loss framing, commitment contracts, cues, or incentives.

## Background

Behavioral economics (BE) is a field at the intersection of psychology and economics that examines why individuals with the goal of maximizing their self-interests engage in unhealthy behaviors they later regret [1]. BE has been used to study several unhealthy behaviors such as overeating or smoking [2–4], but there is little knowledge of how these insights relate to HIV prevention, particularly in African-Americans who have 8.1 times higher rates of HIV infection compared to Whites [5]. Several tools are available to prevent the spread of HIV, including condoms, pre-exposure prophylaxis (PrEP), and testing [6–9]. Yet, uptake of prevention tools depends, in part, on personal choices, and people make sub-optimal choices explained by other temporal or social biases [1, 10]. Despite high HIV burden, 88% of African-Americans reported condomless vaginal sex in the past 12 months (21% condomless anal sex) with low use of HIV testing (44%) and PrEP (0.5%) [11].

This paper presents novel evidence on the prevalence of four common BE biases—present bias, information salience, overoptimism, and loss aversion—in a sample of African-American young adults as it relates to condom use and HIV testing. To our knowledge, this paper is among the first to do so. BE biases have been found to influence health behaviors for other chronic conditions [2–4] and may also be correlated with HIV-related behaviors in racial and ethnic minorities [12–16]. The acute and chronic stress experienced by African-Americans is well documented as a consequence of economic inequalities and discrimination [17, 18]. Research has shown that BE biases are more prominent when individuals experience stress [19–21]. Yet, few studies have examined BE biases in the context of HIV preventive behaviors in vulnerable African-American cohorts.

The first common behavioral bias is *present bias*, which is the tendency of people to yield to current temptation at the expense of future beneficial outcomes [22–24]. For example, a seminal study observed that people repeatedly delayed savings decisions to a future date in exchange for the immediate gratification of spending [25]. This can be challenging for HIV prevention behaviors (e.g., condom use, PrEP, or testing) where the benefits of disease avoidance occur far into the future. Present-biased people may be heavily influenced by immediate benefits of condomless sex (e.g., pleasure and arousal) or the rapid higher earnings from condomless sex and face difficulties carrying out intended condom use when the time comes. We anticipate that young adults with present bias will report lower condom use as their actions may be largely influenced by immediate factors, while discounting long-term goals of avoiding infection.

A second behavioral bias is *information salience*, which is the tendency of people to make decisions based on recent experiences (e.g., what first comes to mind), rather than all available information [26, 27]. For example, individuals often purchase earthquake insurance after a local earthquake given the salience of the experience [28]. Decisions to avoid HIV infection may also be impacted by how salient HIV is in the minds of people and whether memorable events are negative or positive [27, 29, 30]. The health risk of HIV may also be less salient for young adults who know people who are living with HIV—leading to lower use of preventive behaviors [29, 30]. Conversely, HIV may be more salient among those who know someone who has died from AIDS and, consequently, be associated with higher use of preventive behaviors.

*Overoptimism* is a third behavioral bias that may be associated with suboptimal prevention behaviors. It is the tendency to be overly confident in one’s capacity to carry out a planned behavior—and therefore neglect to implement precautions or appropriate steps to stick to the plan [26, 31]. Overoptimism may be expressed in a person’s not purchasing condoms or not discussing condom use in advance. It may also be a factor when a person fails to schedule a clinic visit or obtain documents required for clinic visits (e.g., insurance card). A final potentially-related BE bias is *loss aversion*, a concept in which the threat of loss is a stronger determinant of behavior than the potential to gain [32, 33]. Young adults who are more apt to avoid loss may have higher engagement in preventive behaviors perceived as avoiding loss of sexual health compared to individuals who are less loss averse. Alternatively, they may be more resistant to preventive behaviors perceived to result in loss of affirming sexual relationships.

## Methods

### Aim

This aim of this study was to quantify the prevalence of common behavioral economic biases relating to condom use and HIV testing in young adults with increased likelihood of acquiring HIV and to qualitatively examine young adult’s behavioral and economic perceptions of these biases.

### Design

Data were collected using individual mixed methods interviews within a randomized clinical trial (RCT) intervention called, “*E*ngaging *M*icroenterpris*E* for *R*esource *G*eneration and Health *E*mpowerment” (EMERGE) (clinicaltrials.gov: NCT03766165) for prevention of HIV in economically-vulnerable young adults [34]. We used a single-phase triangulation design in which qualitative questions were embedded into a quantitative survey instrument to validate or expand on the quantitative findings [35]. Data were analyzed with equal weight and interpreted concurrently [35].

### Recruitment and eligibility

Potential participants were recruited on-site from two community-based organizations (CBO) providing emergency and supportive residential services to young adults in Baltimore, Maryland. Baltimore is ranked 19th in HIV infections in the U.S. out of 104 metropolitan statistical areas [44] and has the highest rate of HIV incidence among individuals aged 13 and older than any other jurisdiction in Maryland [45]. Designated CBO staff introduced potential participants to the study team on scheduled visit days. Study eligibility was determined using a screening questionnaire that was administered by a trained interviewer. Individuals were eligible to participate if, at the time of enrollment, they were: African American, aged 18 to 24, living in Baltimore, experiencing homelessness within the last 12 months, unemployed or underemployed (< 30 h per week), out-of-school, and reporting one or more sexual risk behaviors in the last 12 months, such as condomless sex, sex exchange, or sex with a partner of unknown HIV status. Eligible participants were then administered informed consent.

### Data collection

Interviews were conducted in-person by trained interviewers in a private room at the CBO center. Interviewers used a semi-structured interview guide that included quantitative questions with pre-coded categories and subsequent qualitative questions (e.g., no pre-coded responses) that asked participants to explain their pre-coded response. All interviews were conducted in English, audio-recorded, and transcribed. Participants were given snacks and $10 USD in cash after the interview.

### Measures

We measured four behavioral economic biases: present bias, information salience, overoptimism, and loss aversion, using simplified versions of measures applied in other community settings [12, 16, 36–39]. Present bias was assessed using three questions. Participants were given a hypothetical scenario relating to a monetary award, in which they were asked whether they preferred to receive a lottery prize of $1000 USD tomorrow or $3000 USD with a 1 year delay [12, 36]. They were then asked if they had $500 USD whether they were likely to spend all of it or save all of it [16]. They were also given a hypothetical scenario relating to condom use, in which they were asked whether if presented with an opportunity to have sex in which neither sex partner had a condom whether they would wait one day and obtain a condom first or whether they preferred not to wait and proceed with condomless sex [36]. Participants who preferred more immediate outcomes were categorized as present-biased.

Information salience was assessed using four binary questions (e.g., yes/no) regarding whether they knew someone living with HIV, someone who had died of AIDS, someone who had experienced a medical complication due to HIV, or someone who had taken an HIV diagnostic test [12, 37]. They were also asked how often they think about HIV prevention relating to themselves or a sexual partner (e.g., never, rarely, sometimes, often, always) [38]. HIV was considered to be more salient among participants who reported thinking often or always about HIV prevention or among participants who knew someone with an HIV/AIDS-related experience [37, 38].

Overoptimism was examined using three binary questions (e.g., yes/no). Participants were asked whether in the last month, they had planned to use a condom, take an HIV test, or talk to sex partners about protecting against HIV, respectively, but failed in the moment to stick to their decision. Loss aversion was examined with one question in which participants were asked to rate on a scale of 1 to 10 the extent of loss they would encounter if they had a positive HIV test result in the future [39]. Participants reporting higher scores were considered to be more loss averse. After each of the quantitative questions, participants were also asked to provide a qualitative rationale for their selected response, such as a prior experience or reason for their answer. Demographic characteristics relating to age, gender, education, and other socio-economic measures were also obtained.

### Analysis

Descriptive quantitative analyses were conducted using STATA BE (Version 17.0). We examined the itemized distribution of each behavioral economic measure by total and by gender (e.g., male and female). Non-binary and non-gender conforming categories were omitted given that all participants identified as cisgender. Qualitative data were analyzed using Dedoose (Version 9.0.62). We used dual deductive and inductive content analyses. First, we deductively coded responses according to previously identified BE topics in the interview guide. Second, after re-reading participants’ transcripts, we inductively coded responses within each BE topic based on emerging patterns in participants’ explanations. Exemplary quotations were extracted to support findings and labeled with the participant’s gender and age.

## Results

### Sample demographics

A total of 43 young adults were enrolled in the study (Table 1). The mean age of participants was 21.0 years, ranging from age 18 to 24. All (100%) participants identified as African-American. Thirty-five percent (35%) identified as male. Most participants (70%) had a high school diploma or equivalent, although 28% had completed only grades 8 to 11. Unemployment was high (81%) as was the proportion of participants who were income insecure (84%) (e.g., reporting not having enough money to buy food, housing, and/or transportation in the last 30 days). 44% of participants had a bank account in their name, and 19% were parents.

**Table 1:** Demographic and sexual characteristics of enrolled U.S. young adults, aged 18–24, by gender and by total (N = 43)

Characteristic	Gender		Total
	Male	Female	
Number of participants	15	28	43
Proportion of total sample	35%	65%	100%
Mean age in years (± SD)	21.4 (± 1.9)	20.9 ( ±1.5)	21.0 (± 1.6)
Age range in years (min, max)	18, 24	18, 24	18, 24
Highest level of education^a^			
Grades 8 to 11	33%	25%	28%
High school diploma	67%	71%	70%
2 year college	0	4%	2%
4 year college	0	0	0
Unemployed	73%	86%	81%
Income insecurity in last 30 days	93%	79%	84%
Has a banking account	53%	39%	44%
Previous night’s residence^a^			
Emergency shelter (CBO)	0	29%	19%
Transitional housing (CBO)	27%	50%	42%
With friend, relative, partner	60%	18%	33%
With stranger	7%	0	2%
Street/public place	0	0	0
Private apartment	7%	4%	5%
Currently a parent	13%	21%	19%
Had condomless sex in last 30 days	27%	57%	47%
Tested for HIV in last 30 days	53%	61%	58%
Currently taking PrEP	13%	4%	7%

^a^Percentages may not sum to 100% due to rounding

### Sexual and HIV care-seeking behaviors

Half of participants (47%) reported engaging in one or more condomless sex acts without any HIV medications in the last month, while 58% had taken a test for HIV in the last month (Table 1). Uptake of PrEP was low (7%) given participants’ reported behaviors, but in line with overall low levels of PrEP use in the population.

### Present bias

Table 2 describes the prevalence of present bias by gender and total. Approximately half of participants were categorized as present-biased given their preference for $1000 tomorrow (56%) compared to $3000 in one year (44%) (Table 2). 51% also preferred to spend $500 rather than save $500 (49%). For both measures, male participants exhibited higher present bias responses than female participants (60% vs. 54%, respectively; and 53% vs. 50%, respectively), although statistical significance was not assessed. Table 3 provides example quotations of participants’ qualitative explanations for their chosen survey response. The most common explanations for preferring a more immediate reward were needing to address one’s current financial situation and expecting that one could earn a return on their investment more quickly with the immediate and smaller amount (e.g., “flip”). The most common explanations for preferring the more delayed reward were expecting that any money in-hand would be spent unwisely, needing time to determine how to use or invest resources, being able to wait for a larger amount, and being able to wait since not urgently needing money.

**Table 2:** Prevalence of behavioral economic biases of present bias, information salience, overoptimism, and loss aversion in U.S. young adults, aged 18–24 years, by gender and total (N = 43)

Behavioral economic bias	Gender		Total
	Male	Female	
Number of participants	15	28	43
Present bias			
Prefers $1000 USD tomorrow versus $3000 USD in 1 year			
Yes	60%	54%	56%
No	40%	46%	44%
Prefers to spend $500 rather than save $500			
Yes	53%	50%	51%
No	47%	50%	49%
Prefers to have condomless sex than wait for condom			
Yes	33%	25%	28%
No	67%	75%	72%
Information salience			
Knows someone living with HIV			
Yes	27%	39%	35%
No	73%	61%	65%
Knows someone who has died of AIDS^a^			
Yes	27%	29%	28%
No	67%	71%	70%
Knows someone who has had a medical complication due to HIV/AIDS^a^			
Yes	13%	32%	26%
No	80%	64%	70%
Knows someone who has taken an HIV test			
Yes	80%	61%	67%
No	20%	39%	33%
Often or always thinks about HIV prevention			
Yes	67%	79%	74%
No	33%	21%	26%
Overoptimism			
Planned to use condom but found it difficult to stick to decision^a^			
Yes	33%	39%	37%
No	60%	60%	60%
Planned to get tested for HIV but found it difficult to stick to decision^a^			
Yes	13%	7%	9%
No	80%	93%	88%
Planned to talk to partners about HIV but difficult to stick to decision			
Yes	27%	14%	19%
No	73%	86%	81%
Reported over-optimism for ≥ 1 item			
Yes	47%	46%	47%
No	53%	54%	53%
Loss aversion			
Loss encountered from HIV + test result (mean score, ± SD)	9.7 (± 1.3)	9.2 (± 2.1)	9.4 (± 1.9)

^a^Percentages do not add up to 100 due to “don’t know” response

**Table 3:** Example quotations by behavioral economic topic (e.g., present bias, information salience, overoptimism, and loss aversion) from qualitative follow-up with enrolled U.S. young adults (N = 43)

Topic	Survey response [deductive codes]	Example explanatory quotations	Interpretation [inductive codes]
Present bias	Prefer to receive immediate reward	“*Because I am not working. Because of my situation. Now honestly, I would want to get the $3000 in a year and just work, and I know I have that coming. But because of my situation, I would take the $1000 because I need it*.” Male, 24	Needing to address current situation
		“*Because for people who do not have a job, if you got time for the $3000, then you’re going to wait. But if you need money, and depending on your situation, you would take that $1000. It all depends on your situation.”* Male, 23	
		*“$1000 tomorrow. Honestly ‘cause I need money. I got to take care of things, and properly find housing, so…”* Female, 20	
		“*The reason why I say $1000 is that pretty much, what you say, next day, right? Yeah, next day. Um, it’s because … if you were to choose the $3000 you know next year or whatever, you could somehow, you know, flip that $1000 that next day and earn that before the next year comes by.”* Male, 21	Expecting one could earn faster by investing the smaller, immediate amount
		*“I’d take that $1000 any day ‘cause I can turn that $1000 into $3000 within that time, you feel me? Probably more. So, I wouldn’t even need to wait. By the time, I’d probably overlap that three, you know what I mean?”* Male, 22	
	Prefer to receive delayed reward	*“The $3000 in one year…. I might need it a year from now. A thousand would be gone the same day! That’s pretty much it. I get the $1000 tomorrow, it’s gone. I owe some people some money…”* Female, 24	Expecting money in-hand would be spent unwisely
		*“$3000 in a year…. Because one, if I get $1000 in one lump sum, I’m going to spend it*.” Female, 20	
		“*Because, first off, $1000 tomorrow, it would… it sounds good, but realistically speaking, I would run through that money. …Yeah, so if a year from now, I can have $3000, plus me saving up towards the $3000, I can have more than $3000 in a year.”* Female, 21	
		*“Next year, $3000…. Cause if you take for the easy pay tomorrow… That $1000 might go quicker than just waiting on the year to come up with decent ideas of what you are going to do with that money. Or what you want to do with the money, period.”* Male, 23	
		*“Three… um… $3000 in the next year… Oh, I can use that and then I probably, you know, make a plan to have a certain amount of money by that time*” Female, 23	Needing time to determine how to use
		“*I would…a year from now. There’s more money …. I could take that and flip that and make into something else. I can wait, be patient, you can’t always rush into things. That’s how you mess up.”* Female, 22	Able to wait since preferring larger amount
		*“Why would I wait? …You get more money. I just look at it like that. The value is higher, so it’s like, to be patient, for a higher amount of money, sure.”* Female, 22	
		*“It’s more, and it’s like I already lived without it. You know what I mean? So, it wasn’t like it’s something I was expecting anyway, but if it’s, you know. I’m ok with delayed gratification, so…”* Female, 24	
		*“I would say $3000 in one year… ‘cause I ain’t having no $1000 all this time. And, I’m good. So, I can go for another year and get the three. God always said, ‘those who wait’, you know. More come to you. Don’t be greedy. Don’t rush it. Wait on it. And I’ve been doing merry all this time without a $1000, so might as well wait on that good three.”* Male, 24	Able to wait since not urgently in need
	Prefer not to wait for sex with a condom	*“I’m not going to wait. So, if I give them that ultimatum, they be like OK, what are we going to do now? That’s too long. I shouldn’t be this ruthless. I’m really not this promiscuous.”* Male, 24	Perceived excessively long duration
		*“I probably wouldn’t wait. ‘Cause it’s like if I’m into the girl and I’ve been trying to get with her for a while and I finally got the chance to get some and we don’t have a condom. I’m like, ugh, I’m trying to get the girl for years, so I’ll just go ahead and do it.”* Male, 24	Too much when accounting for previous long wait
	Prefer to wait for sex with a condom	“*No. No. I don’t play those games. I’d wait. It ain’t that important. I just like to stay protected. If I don’t got no condom, I can’t do nothing with you. One thing I learned about this area right here is it’s the most highly populated HIV area in Baltimore. You got to definitely watch yourself, period, no matter where you go, but always, always protect yourself.”* Male, 23	Protecting oneself is more important
		*“I’m going to just wait…. I’m going to be honest with you, ‘cause my life is more important. You know, I’ve a friend that unfortunately got caught up in a sticky situation. Her life has changed forever, and um, she’s devastated. And I can’t imagine.”* Female, 22	
		*“I’m not that pressed to have sex. Look, come back [in a few] days. I’m going to holler at you; you feel me? Yes sir.”* Male, 23	Not in urgent need for sex
		*“I would wait… definitely not have sex because I would feel convicted. Like fore you even to bring that up to me, like, I would be like, oh you’re right. You’re right. This is why we shake in those seats when we go for testing. That’s why. That conviction.”* Male, 24	Worry of potential risk
Information salience	HIV is salient	*“Yes, my childhood friend. Yeah, he has been in and out of the hospital multiple times before… I mean, he almost got to the point where he almost had AIDS. You know, because his T-cell count was so high… ‘Cause I’m sure, I know, I know that there isn’t a cure for HIV, but I know there is some very expensive meds to help it be undetected in your body.”* Female, 22	Past acquaintances incurring hospital visits
		*“Um, they got pneumonia. It was really hard for them to shake and um, and sometimes lesions show. And I’ve seen a lot of domestic violence that has come as stem of that. Like, there are a lot of people here that have HIV. Like a lot … and I’ve seen them get beat up that they had this and it was revealed. Um, so it’s a lot…”* Female, 24	Residents experiencing HIV and related violence
		*“One of them is my boyfriend that I’m with now. Yep, and the other one, is my best friend, my godmother, I know a lot of people with HIV. … I already know a lot about it and know like you can live a healthy, happy life, you feel what I’m saying… I already know so much about it.”* Male, 24	Current acquaintances living healthily with HIV
		*“You’d be surprised, but most of the people I know get tested for HIV.”* Female, 22	Social norms among peers
		*“Every time that certain friends of mine have sex with someone, they always want to wear a condom because they are aware of the percentage of how high a person may get an STD from having sex.”* Male, 21	
	HIV is not salient	*“I don’t think if I knew someone that they would tell me that. Because that’s kind of like private.”* Female, 20	Private information not shared with others
Overoptimism	Unable to stick to condom use or HIV test decision	*“Yes. There have been times, OK. I go get the HIV test ‘cause I know that I can be careless at times because I’m in the moment. There have been times where I’m like, “I really want to get tested” and don’t go because I fear the results. You know what I mean*.” Male, 24	Inhibited by fear
		*“Yeah ‘cause when you have it in your mind to do it, but once you do it…, it’s just like you forget all about it.”* Female, 18	Unable to focus on prior plans in the moment
		*“Um, yeah! Yeah, that happened. Oh my goodness… You believe in love at first sight?”* Female, 23	
		“*No, that’s never happened. We live in Baltimore…This is the city of AIDS and HIV. No, too scary. So, umm, no, I mean, even when I was really scared, like last year. Cause I had oral sex with someone last year. I took a note to the nurse in the ER and just, just said, I need this [test]. So yeah…”* Female, 24	Feeling awkward and uncomfortable in the moment
	Able to stick to condom use or HIV test decision	*“Because, like, I don’t have time for no STDs or, you know, things out here are like, a couple things in my life, where I’ve had friends and family, my aunt died from HIV/AIDS. My friend, not my friend, my cousin, she always getting stuff like chlamydia and trichomonas, stuff you can’t get rid of.”* Female, 20	Determined to prevent long-lasting infection
		*“No. Actually when the people came here to HIV test people here, I was the only one who got tested that day.”* Female, 21	Willing to stand alone
		*“No, I always get HIV test. Every time I get a [test]… every 6 months I get an HIV test and a STD test. When I first I started having intercourse, it’s the first thing my mother told me: ‘Every six months, you go get yourself checked’. You don’t need to have social security or nothing. You go to the free clinic on Caroline. By yourself. So yeah…”* Female, 22	Part of health care routine
Loss aversion	High aversion	“*I’d say 10. I’d be very disappointed to find out that I had it, knowing that I’ve had a lot of practice and protected sex.”* Male, 23	Self-blame and regret since having tried to remain negative
		*“I’ve had so many conversations with myself about this. I knew exactly what I was doing while I did it, still did it, and then for that, for something like that to happen, I would check out. I wouldn’t have sex with no one. I wouldn’t be dealing with people because I never wanted to have it and I feel it’s like a consequence for being, um, careless, and I feel like I would just live that punishment out.”* Male, 24	
		*“A 10. I would be very upset. I mean, just because I feel as though I let myself down. You know, and I wasn’t taking the necessary precautions to, you know, stop that from happening.”* Female, 22	
		*“Oh, I would be disappointed, no lie though, I would be hurting. I be losing my mind, honestly. 10. I would be, I would stop talking to people and everything.”* Male, 19	Unable to cope
		*“Very upset. I wouldn’t take it out. The thing is… I’d be very upset, I’d be very disappointed, I’d be very depressed because I never wanted to; nobody ever wants to get it. If I got it, there’s nothing you can do. I would be inconsolable.”* Male, 24	
		*“I would be disappointed in the person that I trusted, like because, when you have sex, there should be trust and if you can’t trust that person, you shouldn’t go to bed with that person. Honestly.”* Female, 20	Broken trust
	Low aversion	“*…I’d have to rethink and look back and say, ‘I know people that got that shit and they still living a healthy, happy life.’ I just got to get my medicine, you know, stay, you know, focused. Learn more about it and shit…*” Male, 24	Still able to live happily

When presented with a hypothetical scenario of delaying sex by 1 day until they were able to acquire a condom, 28% of participants reported that they would prefer immediate condomless sex rather than waiting for a condom, while 72% stated they would be willing to wait for condom-supported sex (Table 2). A third (33%) of male participants were not willing to wait for a condom compared to 25% of female participants. The most common qualitative explanations for not waiting for a condom were the perceived excessively long delay (~ 1 day) and finding the wait too long when accounting for prior time spent to find a sex partner (Table 3). The most common explanations for waiting a day to have condom-supported sex were the importance of protecting against HIV given the high-risk metropolitan area, worrying about the potential risk, and not being in urgent need of sex (Table 3).

### Information salience

HIV salience relating to individuals living with HIV varied among participants. Thirty-five percent (35%) of participants knew someone living with HIV, while 65% did not (Table 2). The majority (70%) also did not know someone who had died of AIDS nor someone who had a medical complication due to HIV/AIDS (70%). In contrast, HIV salience relating to prevention behaviors was higher. Sixty-seven percent (67%) of participants knew someone who had taken an HIV test, and 74% stated they often or always think about preventing HIV (Table 2). HIV salience relating to individuals living with HIV and preventive behaviors appeared to be higher among female participants (39% vs. 27%) with the exception of knowing someone who had tested for HIV (61% vs. 80%). Qualitative examples provided by participants included descriptions of past acquaintances with HIV-related hospitalizations, residents who had experienced HIV-related violence and stigma, and current acquaintances, sex partners, and relatives who were living healthily with HIV (Table 3). They also noted in qualitative responses social norms regarding HIV testing and condom use as behaviors that most residents do given the high prevalence of HIV in Baltimore. The prominent explanation for not knowing someone with an HIV-related experience was that infection status was private information and not readily shared with others (Table 3).

### Overoptimism

Nearly half (47%) of participants reported being overly optimistic one or more times in their intention to protect against HIV (Table 2). Overoptimism appeared to be similar regardless of gender (47% vs. 46%). Among all participants, 37% stated that they planned to use a condom but found it difficult to stick to their decision, compared to 19% and 9% who planned to talk with their sex partner(s) about HIV or take an HIV test, respectively, but found it difficult to stick to their decision (Table 2). For any one HIV preventive behavior, most participants affirmatively reported having little problem sticking to their intended preventive decision (53% to 93%). A common qualitative explanation for being unable to stick to one’s HIV testing decision included being inhibited by fear (Table 3). Common qualitative explanations for being unable to stick to one’s condom use decision included not thinking about condom use in the moment or feeling awkward and uncomfortable to raise the topic in the moment. Among participants who reported being able to stick to their testing and condom use decisions, common qualitative reasons were being determined to prevent long-lasting infection via condoms, knowing someone who had been adversely impacted by HIV, being willing to stand on own’s own, and setting HIV testing as part of their health care routine (Table 3).

### Loss aversion

Loss aversion was high among study participants. The mean score for the extent of loss if one were to have a positive HIV test result was 9.4 (SD + 1.9) (Table 2). Scores ranged from 1 to 10 with higher scores representing higher loss aversion. The median score (50th percentile) for both male and female participants was the maximum score of 10. Only one participant (n = 1, 2.3%) provided a score < 5. Male participants appeared to rate loss related to HIV infection (9.7, SD + 1.3, range: 5–10) similarly to female participants (9.2, SD + 2.1, range: 1–10) (Table 2). Qualitative responses relating to perceived high loss included self-blame and regret since having tried to remain HIV-negative, being unable to cope, and having broken trust (Table 3). The qualitative reason relating to perceived low loss (e.g., score < 5) was knowing other people living healthily and happily with HIV.

## Discussion

This study innovatively aimed to examine the prevalence of four common BE biases—present bias, information salience, overoptimism, and loss aversion—in a sample of African-American young adults as it related to condom use and HIV testing. To our knowledge, this paper is among the first to do so. We found that a mixed methods BE assessment was useful in understanding factors contributing to young adults’ optimal and sub-optimal prevention decisions. Over half of participants exhibited high monetary present bias as may be expected in a population experiencing economic hardship. Much of the driving force of present bias centered on a need to meet minimum financial obligations. As such, HIV prevention programs involving individuals experiencing economic hardship should be mindful of the financial costs associated with promoted preventive behaviors. Coupling income-generating activities, vouchers, financial assistance, or high-yield savings products in addition to health insurance with HIV prevention programs may enable participants to divert more time and resources to HIV risk reduction strategies. This seemed particularly true for participants who justified their preference for an immediate monetary award with an expectation of using current financial assistance to obtain a higher return than would be possible if financial assistance were delayed by a year.

Information salience for HIV was high among participants. Most stated that HIV was often and always on their minds. However, salience related more to thinking about preventive behaviors (e.g., condom use, testing) and the high HIV prevalence in their community rather than thinking about individuals living with HIV or who had experienced a medical complication due to HIV. This could be explained by potential low communication among sex partners and peers of one’s HIV status. Participants suggested in qualitative responses that persons living with HIV experienced high stigma and violence, which may also discourage status disclosure or knowledge of others’ status. Such findings suggest that HIV is a salient topic for urban African-American young adults and that talking about HIV risk reduction would resonate with their ongoing health concerns. However, young adults may relate more to programs focusing on specific prevention behaviors for themselves and their peers than to programs focusing on risks related to uncommon occurrences of acquiring HIV.

Interestingly, despite reporting high information salience for HIV, overoptimism at some point appeared to be a barrier to HIV risk reduction in this population. Nearly half of participants indicated having difficulty carrying out prevention behaviors as intended. This was evident also in the relatively high prevalence of condomless sex in the study sample and relatively low prevalence of HIV testing and PrEP use. This finding suggests that risk reduction programs may need to assist individuals to better carry out their intended preventive behaviors, such as identifying specific barriers, creating action plans, or enlisting supportive peers and cues to remind them of their prevention goals in the moment [40, 41]. Our study found that male participants felt awkward and uncomfortable carrying out condom use intentions with sex partners with whom they hadn’t previously discussed HIV prevention. Carrying out intended condom use also appeared to be difficult for female participants. As such, young adults may benefit from programs that offer strategies on how to talk about male condom use or how to secure female condoms, including when and whether to use PrEP. Providing health insurance or financial incentives for engagement in preventive behaviors may additionally make them financially-attractive to young adults and serve as a nudge for trying a new behavior [27, 42]. HIV risk reduction programs should also target reported fears around coping with potentially positive HIV test results, as this was a common reason provided for omitting testing when the time came.

Yet, an encouraging finding of the study was the high number of affirmative reports by participants of sticking to their intended preventive behavior. This was primarily attributed to being willing to be the lone health advocate, having integrated HIV prevention into their routine health care, or knowing someone adversely impacted by HIV. Participants who preferred to wait for condom use also frequently underscored the importance of protecting themselves at all costs from HIV. Enlisting prevention-focused young adults to support thinking and habit formation among their peers may be a promising peer-mentoring approach [27, 43].

HIV risk reduction efforts might also include assisting present-biased young adults in estimating the financial costs associated with prevention behaviors (e.g., buying condoms, insurance co-pays, clinic travel expenses, etc.). About half of survey respondents acknowledged tendencies to spend rather than save, including spending money swiftly and unwisely. Encouraging youth who are prone to spend to use those resources on HIV prevention may be helpful. In addition, incorporating health savings accounts and other financial products that enable young adults to accumulate earmarked monies for future HIV prevention services may prove valuable for those with these biases.

Finally, while many people are living healthily with HIV, our study found that most young adults were strongly averse to losing their HIV negative status. This may mean that framing messages around loss of status may be more effective than framing messages around gains. Conversely, positively framing messages around maintaining trust in relationships, avoiding regrets, or not letting yourself down may also be beneficial as these were the qualitative reasons quoted by participants who gave high aversion scores [41]. In addition, commitment contracts, such as asking participants to financially pre-commit to a preventive behavior and risk losses if they do not reach their goal, may be a promising tool among participants who are sensitive to the prospect of losing something [27, 40, 42]. Finally, destigmatizing living with HIV may encourage all young adults, whether living with HIV or not, to more openly discuss their status as it relates to primary and secondary prevention intentions.

### Limitations

The limitations of this study should be considered. First, given the study’s small sample and exploratory design, we were unable to assess statistical associations between reported behavioral economic biases and preventive behaviors. Our preliminary quantitative findings should be examined against future studies with more robust statistical samples. Yet, the use of qualitative feedback improved the credibility and robustness of our quantitative data. Second, while the behavioral economic questions used in this analysis were informed by prior studies, there are no standard measures. We purposefully used relatively simple quantitative questions which were followed by explanatory qualitative inquiry. These modified measures were found to be feasible and insightful. However, more research is needed to assess the validity and reliability of these and more complex BE measures in vulnerable African-American young adults. Finally, all responses were based on self-report and may have been subject to social desirability biases. However, despite these limitations, the study had several strengths. These included its focus on an understudied topic in racial minority young adults, inclusion of mixed methods data, inclusion of sexual and HIV care-seeking behaviors, and exploration of differences by gender.

## Conclusions

Despite application to numerous health conditions, behavioral economic biases relating to HIV prevention in vulnerable African-American young adults are understudied. We found that a mixed methods BE assessment was useful in understanding factors contributing to young adults’ optimal and sub-optimal prevention decisions. Future interventions may benefit from integrating savings products, loss framing, commitment contracts, cues, or incentives into HIV prevention strategies.

## Data Availability

The dataset analyzed during the current study includes qualitative transcripts and notes and is therefore not publicly available due to its containing information that could compromise individual privacy.

## Abbreviations

AIDS: Acquired Immune Deficiency Syndrome
BE: Behavioral Economics
CBO: Community-based Organization
HIV: Human Immunodeficiency Virus
PrEP: Pre-exposure Prophylaxis
SD: Standard Deviation
USD: United States Dollar

## References

[1] Thaler RH and Mullainathan S. How behavioral economics differs from traditional economics. The concise encyclopedia of economics. 2nd ed. behavioral economics. 2008 http://www.econlib.org/library/Enc/BehavioralEconomics.html. Accessed 10 Sep 2022

[2] Heil S, Higgins S, Bernstein I (2008). Effects of voucher-based incentives on abstinence from cigarette smoking and fetal growth among pregnant women. Addiction.

[3] Barsky R, Juster T, Kimball M, Shapiro M (1997). Preference parameters and behavioral heterogeneity: an experimental approach in the health and retirement study. Q J Econ.

[4] Rice T (2013). The behavioral economics of health and health care. Annu Rev Publ Health.

[5] Centers for Disease Control and Prevention (CDC). HIV Surveillance Report, Diagnoses of HIV Infection in the United States and Dependent Areas, 2020; vol. 33. Published May 2022. https://www.cdc.gov/hiv/pdf/library/reports/surveillance/cdc-hiv-surveillance-report-2020-updated-vol-33.pdf. Accessed 11 Dec 2022

[6] Padian NS, Buvé A, Balkus J, Serwadda D, Cates W (2008). Biomedical interventions to prevent HIV infection: evidence, challenges, and way forward. Lancet.

[7] Abbas UL (2011). Uptake of biomedical interventions for prevention of sexually transmitted HIV. Curr Opin HIV AIDS.

[8] Center for Disease Control and Prevention (CDC): Preventing New HIV Infections—Condom Use. Division of HIV/AIDS Prevention. 2020. https://www.cdc.gov/hiv/clinicians/prevention/condoms.html. Accessed 27 Oct 2022

[9] Center for Disease Control and Prevention (CDC): Treatment, Care, and Prevention for People with HIV—Safer Sexual Behavior. Division of HIV/AIDS Prevention. 2019. https://www.cdc.gov/hiv/clinicians/treatment/safer-sex.html. Accessed 28 Oct 2022

[10] Ariely D (2008). Predictably irrational.

[11] Centers for Disease Control and Prevention (CDC). HIV Infection, Risk, Prevention, and Testing Behaviors Among Heterosexually Active Adults at Increased Risk for HIV Infection—National HIV Behavioral Surveillance, 23 U.S. Cities, 2019. HIV Surveillance Special Report 26. Published January 2021. https://www.cdc.gov/hiv/pdf/library/reports/surveillance/cdc-hiv-surveillance-special-report-number-26.pdf. Accessed 03 Nov 2022

[12] Linnemayr S, Stecher C (2015). Behavioral economics matters for HIV research: the impact of behavioral biases on adherence to antiretrovirals (ARVs). AIDS Behav.

[13] Linnemayr S, MacCarthy S, Wagner Z, Barreras JL, Galvan FH (2018). Using behavioral economics to promote HIV prevention for Key populations. J AIDS Clin Res.

[14] Linnemayr S (2015). HIV prevention through the lens of behavioral economics. J Acquir Immune Defic Syndr.

[15] Jennings L, Mathai M, Linnemayr S, Trujillo A, Mak'anyengo M, Montgomery BEE, Kerrigan DL (2017). Economic context and HIV vulnerability in adolescents and young adults living in urban slums in Kenya: a qualitative analysis based on scarcity theory. AIDS Behav.

[16] Mayo-Wilson LJ, Ssewamala FM (2019). Financial and behavioral economic factors associated with HIV testing in AIDS-affected adolescents in Uganda: a cross-sectional analysis. J Health Care Poor Underserved.

[17] McKenzie K (2003). Racism and health: antiracism is an important health issue. BMJ.

[18] Gee GC, Ford CL (2011). Structural racism and health inequities: old issues and new directions. Du Bois Rev.

[19] Haushofer J, Fehr E (2014). On the psychology of poverty. Science.

[20] Mani A, Mullainathan S, Shafir E, Zhao J (2013). Poverty impedes cognitive function. Science.

[21] Mullainathan S, Shafir E (2013). Scarcity: the new science of having less and how it defines our lives.

[22] Frederick S, Loewenstein G, Donoghue O (2002). Time discounting and preference: a critical time review. J Econ Lit.

[23] O’Donoghue T, Rabin M (1999). Doing it now or later. Am Econ Rev.

[24] White JS, Dow WH (2015). Intertemporal choices for health. Behavioral Economics and Public Health..

[25] Benartzi S (2012). Save more tomorrow.

[26] Kahneman D (2011). Thinking, fast and slow.

[27] Taylor NK, Buttenheim AM (2013). Improving utilization of and retention in PMTCT services: can behavioral economics help?. BMC Health Serv Res.

[28] Palm R, Hodgson M. After a California Earthquake: attitude and behavior change. The University of Chicago Geography Research Paper No 233. 1992

[29] Zimmermann HM, van Bilsen WP, Boyd A, Prins M, van Harreveld F, Davidovich U (2021). HIV transmission elimination team amsterdam. prevention challenges with current perceptions of HIV burden among HIV-negative and never-tested men who have sex with men in the Netherlands: a mixed-methods study. J Int AIDS Soc.

[30] van der Snoek EM, de Wit JB, Mulder PG, van der Meijden WI (2005). Incidence of sexually transmitted diseases and HIV infection related to perceived HIV/AIDS threat since highly active antiretroviral therapy availability in men who have sex with men. Sex Transm Dis.

[31] Moore DA, Healy PJ (2008). The trouble with overconfidence. Psychol Rev.

[32] Thaler RH, Tversky A, Kahneman D, Schwartz A (1997). The effect of myopia and loss aversion on risk taking: an experimental test. Quar J Eco.

[33] Chivers LL, Higgins ST (2012). Some observations from behavioral economics for consideration in promoting money management among those with substance use disorders. Am J Drug Alcohol Abuse.

[34] Mayo-Wilson LJ, Glass NE, Ssewamala FM, Linnemayr S, Coleman J, Timbo F, Johnson MW, Davoust M, Labrique A, Yenokyan G, Dodge B, Latkin C (2019). Microenterprise intervention to reduce sexual risk behaviors and increase employment and HIV preventive practices in economically-vulnerable African-American young adults (EMERGE): protocol for a feasibility randomized clinical trial. Trials.

[35] Creswell JW, Plano Clark VL, Creswell JW, Clark VLP (2007). Choosing a mixed methods design. Designing and conducting mixed methods research.

[36] Sweeney MM, Berry MS, Johnson PS, Herrmann ES, Meredith SE, Johnson MW (2020). Demographic and sexual risk predictors of delay discounting of condom-protected sex. Psychol Health.

[37] Grover KW, Miller CT (2014). Effects of mortality salience and perceived vulnerability on HIV testing intentions and behaviour. Psychol Health.

[38] Sewell WC, Patel RR, Blankenship S, Marcus JL, Krakower DS, Chan PA, Parker K (2020). Associations among HIV risk perception, sexual health efficacy, and intent to use PrEP among women: an application of the Risk Perception Attitude Framework. AIDS Educ Prev.

[39] Chard AN, Metheny N, Stephenson R (2017). Perceptions of HIV seriousness, risk, and threat among online samples of HIV-negative men who have sex with men in seven countries. JMIR Public Health Surveill.

[40] Thorgeirsson T, Kawachi I (2013). Behavioral economics: merging psychology and economics for lifestyle interventions. Am J Prev Med.

[41] Mogler BK, Shu SB, Fox CR, Goldstein NJ, Victor RG, Escarce JJ, Shapiro MF (2013). Using insights from behavioral economics and social psychology to help patients manage chronic diseases. J Gen Intern Med.

[42] Linnemayr S, Rice T (2016). Insights from behavioral economics to design more effective incentives for improving chronic health behaviors, with an application to adherence to antiretrovirals. J Acquir Immune Defic Syndr.

[43] Persell SD, Friedberg MW, Meeker D, Linder JA, Fox CR, Goldstein NJ, Shah PD, Knight TK, Doctor JN (2013). Use of behavioral economics and social psychology to improve treatment of acute respiratory infections (BEARI): rationale and design of a cluster randomized controlled trial [1RC4AG039115-01]-study protocol and baseline practice and provider characteristics. BMC Infect Dis.

[44] Centers for Disease Control and Prevention (CDC). Diagnoses of HIV infection among adults and adolescents in metropolitan statistical areas—United States and Puerto Rico, 2018. HIV Surveillance Data Tables 2020;1(No. 3). https://www.cdc.gov/hiv/pdf/library/reports/surveillance-data-tables/vol-1-no-3/cdc-hiv-surveillance-tables-vol-1-no-3.pdf. Accessed 05 Jan 2023

[45] Centers for Disease Control and Prevention (CDC). Estimated HIV incidence and prevalence in the United States, 2015–2019. HIV Surveillance Supplemental Report 2021;26(No. 1). https://www.cdc.gov/hiv/pdf/library/reports/surveillance/cdc-hiv-surveillance-supplemental-report-vol-26-1.pdf. Accessed 19 Dec 2022

